# Use and Evaluation of Computerized Clinical Decision Support Systems for Early Detection of Sepsis in Hospitals: Protocol for a Scoping Review

**DOI:** 10.2196/24899

**Published:** 2020-11-20

**Authors:** Ling Li, Khalia Ackermann, Jannah Baker, Johanna Westbrook

**Affiliations:** 1 Centre for Health Systems and Safety Research Australian Institute of Health Innovation Macquarie University North Ryde Australia

**Keywords:** sepsis, early detection of disease, computerized clinical decision support systems, patient safety, electronic health records, sepsis care pathway

## Abstract

**Background:**

Sepsis is a leading cause of death in hospitals, with high associated costs for both patients and health care systems worldwide. Early detection followed by timely intervention is critical for successful sepsis management and, hence, can save lives. Health care institutions are increasingly leveraging clinical data captured in electronic health records for the development of *computerized clinical decision support* (CCDS) systems aimed at enhancing the early detection of sepsis. However, a comprehensive evidence base regarding sepsis CCDS systems to inform clinical practice, research, and policy is currently lacking.

**Objective:**

This scoping review aims to systematically describe studies reporting on the use and evaluation of CCDS systems for early detection of sepsis in hospitals.

**Methods:**

The methodology for conducting scoping reviews presented by the Joanna Briggs Institute Reviewer’s Manual and the PRISMA-ScR (Preferred Reporting Items for Systematic Reviews and Meta-Analyses extension for Scoping Reviews) will be used and adapted as guides. A comprehensive literature search of 10 electronic databases will be conducted to identify all empirical quantitative and qualitative studies that investigate the use of CCDS systems for early detection of sepsis in hospitals. Detailed inclusion and exclusion criteria have been developed. Two reviewers will independently screen all articles based on these criteria. Any discrepancies will be resolved through discussion and further review by a third researcher if required.

**Results:**

Electronic database searches have retrieved 12,139 references after removing 10,051 duplicates. As of the submission date of this protocol, we have completed the title and abstract screening. A total of 372 references will be included for full-text screening. Only 15.9% (59/372) of these studies were focused on children: 11.0% (41/372) for pediatric and 4.8% (18/372) for neonatal patients. The scoping review and the manuscript will be completed by December 2020.

**Conclusions:**

Results of this review will guide researchers in determining gaps and shortcomings in the current evidence base for CCDS system use and evaluation in the early detection of sepsis. The findings will be shared with key stakeholders in clinical care, research, policy, and patient advocacy.

**International Registered Report Identifier (IRRID):**

PRR1-10.2196/24899

## Introduction

### Sepsis and Early Detection

Sepsis, defined as “life-threatening organ dysfunction caused by a dysregulated host response to infection” [1], is estimated to affect 50 million people each year worldwide, of which more than 40% are among children younger than 5 years [2]. Despite advances in vaccines, antibiotics, and acute care, sepsis remains the leading cause of death from infection [1]. About 20% of patients with sepsis die, with global estimates recording 11 million deaths due to sepsis in 2017 alone [2]. Surviving sepsis is associated with increased mortality across an individual’s lifespan and significant reductions in quality of life, including higher rates of chronic illness, physical disability, cognitive impairment, and mental health issues [3-9]. Additionally, sepsis treatment is extremely expensive [10]. In the United States it has been listed as the most expensive condition in US hospitals (>US $20 billion annually) [11]. The World Health Organization has declared sepsis a global medical emergency and adopted a resolution in 2017 to reduce the global burden of sepsis by improving sepsis diagnosis, treatment, and management [12].

Early detection of sepsis allows for prompt treatment, which is associated with reduced mortality and lower costs [13,14]. The 2016 Surviving Sepsis Campaign (SSC) guidelines, a set of clinical guidelines designed by a panel of international sepsis experts, strongly recommend treatment begin immediately, with the administration of intravenous antimicrobials within 1 hour of sepsis or septic shock recognition [15]. To enhance the effectiveness of rapid treatment, it is critical that septic patients are identified as early as possible [15-17]. Interventions such as regular monitoring of vital signs and elevated lactate levels can aid early recognition [18]. However, studies show that delays in both disease diagnosis and treatment are not uncommon in hospitals [19-21]. One of the main barriers to early sepsis diagnosis is the lack of effective diagnostic tools, further compounded by the fact that sepsis is a heterogeneous and enigmatic syndrome with no diagnostic gold standard [22]. Consequently, clinicians face a challenge in differentiating sepsis from other acute conditions with similar signs or symptoms.

Sepsis detection and recognition pathways can play an important role in facilitating early sepsis diagnosis and initiation of treatment. A number of sepsis-risk warning tools have been developed, for example, the Quick Sepsis-Related Organ Failure Assessment, the National Early Warning Score in the United Kingdom, and the Adult Sepsis Pathway in Australia [23-25]. Many hospitals currently rely upon paper-based sepsis recognition tools, which are susceptible to transcription and interpretation errors and highly reliant upon vigilant and timely patient monitoring by clinicians. In contrast, appropriately designed automated systems have the potential to decrease delays and increase the accuracy of sepsis detection [26].

### Computerized Clinical Decision Support Systems

Given the difficulties associated with timely sepsis recognition, health care institutions are increasingly leveraging clinical data captured in electronic health records (EHRs), which have been rolled out extensively in recent years around the world. EHR-based computerized clinical decision support (CCDS) systems, built into hospital electronic systems, present a great opportunity to facilitate sepsis early detection and prompt treatment. CCDS systems automate sepsis-risk warning tools to alert clinicians to the possible presence of sepsis [16,27], while reducing the mental load on clinicians and nurses [28,29]. Following an alert, a protocol is followed that usually involves a patient being evaluated by senior medical staff to confirm diagnosis and initiate the appropriate sepsis treatment [27].

Over the past 10 years, two distinct CCDS approaches to sepsis identification have emerged: knowledge-based electronic CCDS following predefined *rules* of an established diagnosis pathway, and nonknowledge-based CCDS utilizing artificial intelligence and machine learning techniques [28,30]. Our primary focus in this scoping review is the use of knowledge-based electronic CCDSs in sepsis detection.

### Research Questions and Aims

Implementation of sepsis CCDS in hospital clinical information systems is a novel and rapidly expanding area [28]. Furthermore, the use of technological innovations in health care is rife with complexity [31]. This is particularly true for the use and evaluation of sepsis CCDS in real-world clinical settings. Sepsis itself is a complex and multifaceted condition, and various clinical criteria for sepsis early detection have been developed over the years and implemented in an evolving range of sepsis CCDS systems [32]. These systems have been evaluated in numerous clinical settings in hospitals, such as emergency departments, intensive care units (ICUs), and various wards [32,33]. In addition, there is extensive heterogeneity in the design of studies evaluating the effectiveness of these CCDS systems, such as differing outcome measures (eg, mortality, cost, and clinical workflow) and evaluation methods [28,32]. We intend to explore the complexity present in the use and evaluation of sepsis CCDS by systematically mapping the breadth of the literature in a scoping review to identify knowledge gaps and inform future research. The research question we have formulated for this review is as follows: *What is the evidence base for the use of knowledge-based CCDS systems in hospitals for sepsis early detection and how have they been evaluated?*

In this scoping review, we will aim to (1) scope the study contexts, designs, and research methods employed, (2) summarize study outcomes investigated and outcome measures utilized, and (3) map out the range of CCDS designs and implementation features, such as sepsis clinical criteria and related sepsis care and management protocols.

## Methods

### Scoping Review Method

The methodology for conducting scoping reviews presented by the Joanna Briggs Institute (JBI) Reviewer’s Manual [34] and the PRISMA-ScR (Preferred Reporting Items for Systematic Reviews and Meta-Analyses extension for Scoping Reviews) [35] will be used and adapted as guides. In particular, the five-stage scoping review framework presented by Arksey and O’Malley [36] will be followed as recommended by JBI [35]: (1) identifying the research question, (2) identifying relevant studies, (3) selecting studies, (4) charting the data, and (5) summarizing and reporting the results.

A search for current reviews and protocols on this topic was undertaken on MEDLINE, the JBI database, and Google Scholar, confirming the absence of scoping reviews in this research area. Only one similar scoping review protocol was identified [37]; however, the authors are focusing only on the use of machine learning or artificial intelligence–based CCDS, whereas our review is instead focused on only knowledge-based (ie, nonmachine learning) CCDS.

### Scoping Review Stages

#### Identifying the Research Question

An ideal question for a scoping review is broad, with clear links to the rationale and intended purpose of the review [38,39]. The research question, aims, rationale, and purpose of this review are detailed above. The research question was formulated following preliminary reading and exploratory searches of the literature on CCDS systems for early detection of sepsis and subsequent discussions with the review team. The question, as described above in the Introduction section, was developed in an iterative manner, following contemplation of the rationale and the intended purpose of the research.

#### Identifying Relevant Studies

The search used in a scoping review should be as broad as feasibly possible to ensure a comprehensive overview of the field [36]. To achieve this, we employed a three-step search strategy. The design and refinement of the search strategy was undertaken with consultation from an experienced librarian. During step 1, MEDLINE and CINAHL were searched using a preliminary search strategy derived from the initial exploratory literature searches. The index terms and text words in the abstract and title of the preliminary search results were then analyzed to identify relevant text words and subheading terms. These identified text words and index terms were added to the preliminary search. MEDLINE and Embase were then used to pilot and refine the search strategy to ensure the final search would be as comprehensive as possible, without becoming too time-consuming and impractical. The search strategies of previous relevant systematic reviews were also retrieved and analyzed for additional relevant terms and text words [16,27,28,33,40]. An example of the final search strategy, used to search MEDLINE, is presented in Multimedia Appendix 1. In step 2, all included databases were then searched using the final search strategy. Both peer-reviewed and grey literature databases were searched. During step 3, the reference lists of relevant systematic reviews and of salient papers selected for data charting were hand-searched to identify additional relevant articles.

The databases we intend to search are as follows: MEDLINE; Embase; CINAHL; the Cochrane database, including CENTRAL (Cochrane Central Register of Controlled Trials); LILACS (Latin American and Caribbean Health Sciences Literature); Scopus; Web of Science; OpenGrey; ClinicalTrials.gov; and ProQuest Dissertations and Theses Global. These databases were chosen to ensure that a broad overview of both the black and grey literature relevant to our topic was retrieved by our search. There will be no limits set on publication date and, thus, we will include studies from the inception of each database, up until the date of our search.

#### Selecting Studies

All identified articles will be exported into an EndNote X9 (Clarivate) library, which will be used to manage reference data throughout the review, and duplicates will be removed. Two reviewers will then independently screen the titles and abstracts for relevant articles as determined by the eligibility criteria, with any disagreements resolved through discussion or further review by a third researcher if necessary. The full texts of included articles will then be retrieved and similarly screened by the same two independent reviewers using the eligibility criteria to select the final articles for inclusion. Any disagreements will be resolved though discussion or consultation with a third reviewer. The reference lists of relevant systematic reviews as well as of salient articles selected for inclusion will be hand-searched to identify any further articles. A PRISMA (Preferred Reporting Items for Systematic Reviews and Meta-Analyses) flow diagram will be used to visually illustrate this process.

Title and abstract screening using the eligibility criteria (see Textbox 1 [1,27,30]) will be trialed with a random selection of 25 articles by both reviewers, and discussed with a third reviewer, to ensure that there is consensus within the review team surrounding what is considered to meet the inclusion or exclusion criteria. Once consensus and clarification have been reached, then the search selection will begin. Similarly, once title and abstract screening is completed, full-text screening will be piloted with a random selection of 10 articles to ensure consensus. The study selection process will be an iterative process, with any potential changes to the eligibility criteria or study selection detailed in the final report.

**Eligibility criteria for articles.**

Inclusion criteria (if they met all of the following criteria):

1. Investigated or evaluated a knowledge-based computerized clinical decision support (CCDS) system used for early detection of sepsis. Knowledge-based CCDS systems are those where the algorithm receives, collects, and integrates data to evaluate a predefined rule and then executes the appropriate action [30]. In practice, this means they are programmed with a set of sepsis detection criteria predetermined by humans [27]. Once a patient is calculated to meet these criteria, an action will commence, normally in the form of a sepsis alert [27]. Due to the evolving nature of the official sepsis definition, the most recent of which was only released in 2016 [1], and the intentionally broad scope of this review, we will include all studies that include CCDS systems designed for systemic inflammatory response syndrome (SIRS), sepsis, or septic shock, by any definition.
2. Investigated in any hospital setting. We will include all articles that investigate the use of CCDS in a hospital setting, including but not limited to CCDSs implemented in the emergency department, intensive care units, or in general wards.
3. Any human patient population with sepsis. Studies investigating the use of CCDS for sepsis in any human patient population will be eligible, regardless of age, sex, ethnicity, or comorbidities.
4. Original research investigated the use of an implemented CCDS system. We will include all study designs, provided the studies involve original research evaluating a CCDS that has been implemented in clinical settings.
5. Published in the English language. We will limit our search to only studies published in the English language due to time and resource constraints.

Exclusion criteria (if they met one or more of the following criteria):

1. Investigated the use of a nonknowledge-based CCDS system. A nonknowledge-based CCDS system is one that utilizes machine learning, artificial intelligence, or statistical-based pattern recognition [30]. In this case, rather than relying on set criteria programmed by a human, normally a clinician or researcher, the algorithm is programmed to use advanced computational techniques to independently determine the appropriate action following training on a model data set [30].
2. Only investigated CCDS systems or sepsis, not both. Throughout hospitals, CCDS systems are used for an extensive variety of purposes, including but not limited to disease detection, checking order sets, and improving documentation and communication [30]. Studies will be excluded if the primary focus is not on the detection of sepsis, septic shock, or SIRS, by any definition. There is an extensive body of literature on sepsis itself, relating to a diverse range of subtopics. We will exclude any articles that do not investigate the use of a CCDS system.
3. Simulations of CCDS use, or CCDS use outside of hospitals. We will exclude any studies in which the CCDS system is not implemented in hospitals in the real world, including but not limited to studies in which the CCDS system is only evaluated through a data-driven simulation or is implemented outside of a hospital, such as in prehospital care.
4. Studies that did not include original research on CCDS system use. We will exclude any studies that do not investigate original research on CCDS system use, such as commentaries, editorials, opinion pieces, and reviews. However, while reviews will not be used for data charting or analysis, they may be retained for the purpose of reference list searching for relevant articles.
5. Studies performed in animals or other nonhuman organisms. We will exclude any studies that are not exclusively performed in human populations, such as mouse, dog, or guinea pig studies.

#### Charting the Data

Data charting will be performed by one reviewer using a predesigned charting form, and a second reviewer will double-check the accuracy of a random sample. Any disagreements will be resolved through discussion or consultation with a third reviewer. The data-charting form will be designed in Microsoft Access and initially piloted by the review team to ensure that appropriate and sufficient data are extracted. As discussed by Levac et al [39], data charting is often an iterative and continually updated process; thus, our data-charting form will be altered during extraction as needed, with all changes recorded and explained. The following data will be charted to address the study aims:

1. Study context, design, and research methods (Aim 1). We will extract relevant contextual information, such as authors, the year of publication, CCDS implementation if available, the study title and objectives, and the country of the CCDS implementation. In addition, we will also collect the study setting, defined as the specific situation in which the CCDS is implemented (eg, ICU), and demographic and clinical information of the study population, such as age category (ie, adult, pediatric, neonatal, or all), the numerical age range if given, gender, and any underlying conditions reported to be common to the study population. We will also extract all relevant information about the study design, including the study type, using typical categories as described by Ranganathan and Aggarwal [41-45]; how sepsis patients were identified (eg, chart review or EHR data); and study power, if relevant.
2. Study outcomes and outcome measurements (Aim 2). We will extract all relevant information about all outcomes investigated, including outcome category, which is predecided by the research team (ie, patient outcomes, sepsis treatment and management, CCDS usability, and cost); the specific outcome itself; and the outcome measures used. The patient outcome category is defined as outcomes that directly measure the change in patient health care endpoints, such as mortality, ICU admission, and hospital length of stay. The prognostic accuracy of the CCDS systems in predicting these outcomes could be reported using measurements such as sensitivity, specificity, positive predictive value, negative predictive value, and area under receiver operating characteristic curve. The sepsis treatment and management category is defined as outcomes that measure the change in sepsis management following CCDS implementation, such as time to diagnosis, time to treatment, and SSC guideline adherence. The usability category is defined as outcomes that are related to the usability or user experience of CCDS, such as clinician perceptions. The cost category is defined as outcomes that are related to the cost or cost-effectiveness of CCDS implementation and use.
3. CCDS design and implementation features (Aim 3). We will extract all relevant information regarding the design of the CCDS, such as CCDS type (ie, either commercial or homegrown), and details regarding the implementation of the CCDS, such as the type of responding personnel, the type of alert, and clinical integration method. Commercial CCDS systems are defined as CCDS systems that have been purchased from an external supplier. Some commercial CCDS systems may have been subsequently modified; however, for the purpose of this review, we will still consider them as commercial. Homegrown CCDS systems are defined as CCDS systems that have been designed in-house by the institution implementing it. The method of clinical integration of CCDS will include information regarding where the CCDS has been implemented, how it works, whether response teams and specific care and management protocols or bundles were simultaneously introduced, and what the vital sign criteria are if available.

#### Summarizing and Reporting the Results

The results will be analyzed through both a narrative review and quantitative analysis. A narrative summary of the data will be presented, organized by our three aims. Each aim may be further divided into sections, which will be determined iteratively following data charting. Basic summary statistics, primarily frequency counts and percentages, will also be used to give a numerical overview of the data. The data will be presented in tabular and graphical forms to support the narrative review, and numerical analysis and will be designed iteratively following data charting with consideration for the intended purpose of the review.

The results may be divided and published over multiple papers, depending on the quantity of data charted. If this is considered, the split will be a well-documented iterative process. It is likely that we will either publish two papers divided by population age category, in which we may publish the results for adult populations and pediatric populations separately, or we may publish a second paper focusing only on mapping CCDS type and design across the literature. This will be determined following data charting.

### Ethics

Ethical approval or consent to participate is not required for this protocol and scoping review. The data will be extracted from published articles, and no individual information will be included.

## Results

**Figure 1:**
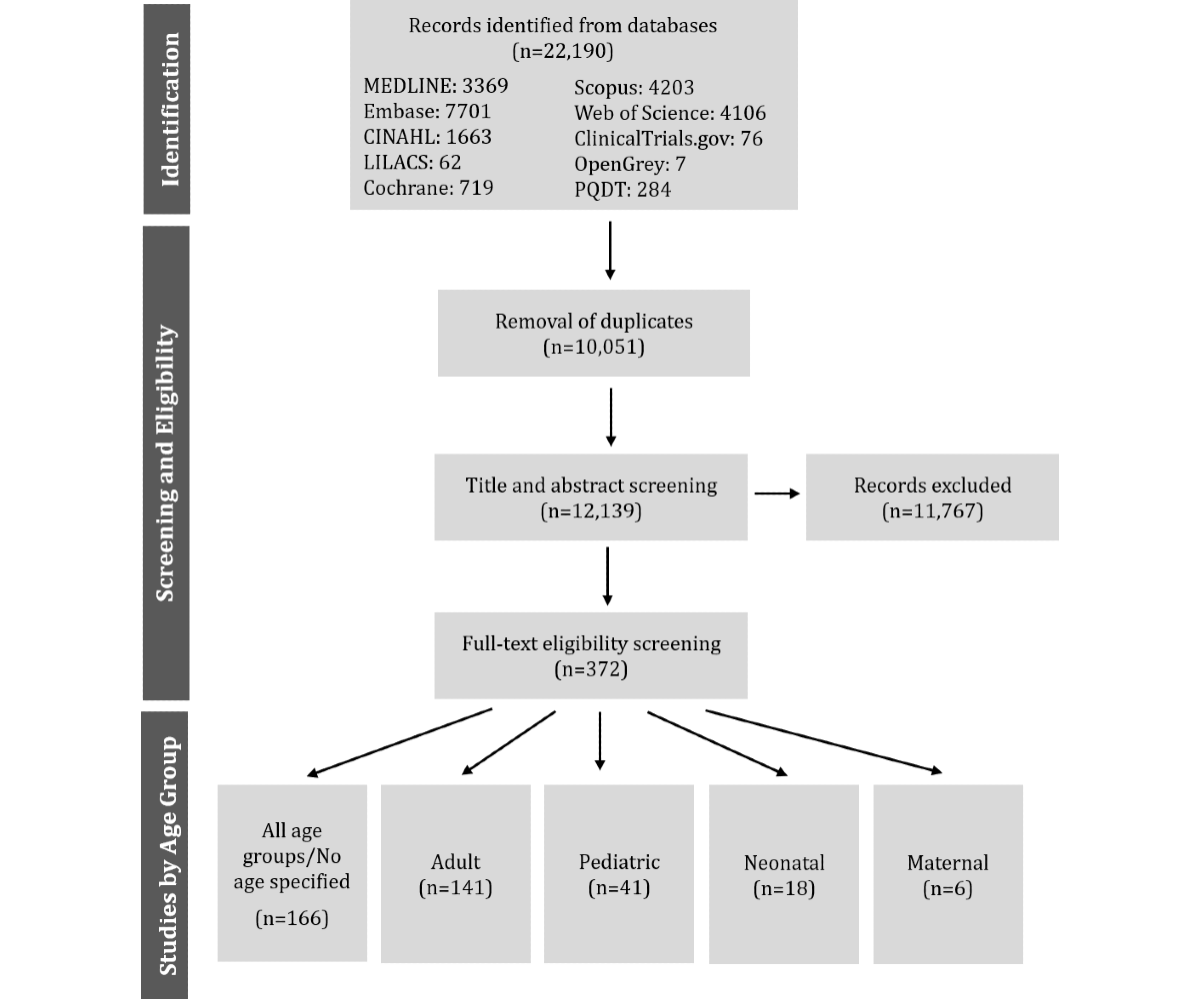
Flowchart of the preliminary search results and screening process. LILACS: Latin American and Caribbean Health Sciences Literature; PQDT: ProQuest Dissertations and Theses.

As of the submission date of this protocol, title and abstract screening has been completed. Figure 1 illustrates the search results and screening process. The search was run in September 2020 and resulted in 22,190 references. After removing 10,051 duplicates, 12,139 references were included for title and abstract screening by two reviewers. The full texts of 372 references will be screened for inclusion in the final review. A total of 44.6% (166/372) of these references included all age groups or did not specify age, another 37.9% (141/372) were focused on adult patients, and the rest were for pediatric (41/372, 11.0%), neonatal (18/372, 4.8%), and maternal patients (6/372, 1.6%). Data charting and analysis will follow with the aim to submit a manuscript describing initial results by the end of December 2020.

## Discussion

### Overview

The increasing digitization of health care has promoted the use of CCDS systems in hospitals for sepsis early detection and treatment [27]. However, there is poor consensus on the effectiveness of these systems in improving the health outcomes of patients with sepsis [28]. This can be partly attributed to the complex and heterogenic nature of studies investigating this topic [16,28]. Additionally, as the field is rapidly emerging, there are increasingly varied methods of CCDS evaluation seen in the literature. In this paper, we have presented a protocol for a scoping review based on well-established methodology, as explained in the Methods section. A strength of our review, and of scoping reviews in general, is their broad search strategy and eligibility criteria, which will allow us to scope the breadth of the field more comprehensively. To our knowledge, this scoping review is the first review to comprehensively map the breadth of the literature investigating the use of CCDS for the early detection of sepsis in hospitals. Mapping the literature will provide a broad outline of the current studies within the field, promoting a more comprehensive understanding of current research efforts. We will, therefore, be better able to identify research gaps, which in turn can inform future studies and clinical practice.

Due to feasibility and time constraints, we limited our search to studies published in English, or that had English translations readily available. Therefore, we must acknowledge that our scoping review may miss some studies published only in non-English languages.

### Conclusions

The review will provide a comprehensive summary on the use of knowledge-based CCDS systems in the early detection of sepsis in hospitals, providing researchers, clinicians, policy makers, and developers with a relevant and important evidence base. Our findings will highlight research gaps and shortcomings in existing evaluations and implementations of sepsis CCDS and, hence, guide future research efforts. The results will be shared with key stakeholders in clinical care, research, policy, and patient advocacy to inform clinical practice and policy.

## Appendix

Multimedia Appendix 1 MEDLINE search strategy.

## Abbreviations

CCDS: computerized clinical decision support
CENTRAL: Cochrane Central Register of Controlled Trials
EHR: electronic health record
ICU: intensive care unit
JBI: Joanna Briggs Institute
LILACS: Latin American and Caribbean Health Sciences Literature
PRISMA: Preferred Reporting Items for Systematic Reviews and Meta-Analyses
PRISMA-ScR: Preferred Reporting Items for Systematic Reviews and Meta-Analyses extension for Scoping Reviews
SSC: Surviving Sepsis Campaign
